# Ex Vivo and In Vivo Analyses of Novel 3D-Printed Bone Substitute Scaffolds Incorporating Biphasic Calcium Phosphate Granules for Bone Regeneration

**DOI:** 10.3390/ijms22073588

**Published:** 2021-03-30

**Authors:** Franciska Oberdiek, Carlos Ivan Vargas, Patrick Rider, Milijana Batinic, Oliver Görke, Milena Radenković, Stevo Najman, Jose Manuel Baena, Ole Jung, Mike Barbeck

**Affiliations:** 1 ScientiFY GmbH, 15806 Zossen, Germanypatrick.rider@botiss.com (P.R.); 2 Escuela Técnica Superior de Ingenieros Industriales, Universidad Politécnica de Madrid, Calle José Gutierrez Abascal, 2, 28006 Madrid, Spain; 3 REGEMAT 3D, Avenida del conocimiento 41, A-111, 18016 Granada, Spain; ceo@regemat3d.com; 4 Research Department, BerlinAnalytix GmbH, 12109 Berlin, Germany; milijana.batinic@berlinanalytix.com; 5 Department of Ceramic Materials, Chair of Advanced Ceramic Materials, Institute for Materials Science and Technologies, Technical University Berlin, 10623 Berlin, Germany; o.goerke@tu-berlin.de; 6 Scientific Research Center for Biomedicine, Department for Cell and Tissue Engineering, Faculty of Medicine, University of Niš, 18000 Niš, Serbia; milena1390nis@gmail.com (M.R.); stevo.najman@gmail.com (S.N.); 7 Department of Biology and Human Genetics, Faculty of Medicine, University of Niš, 18000 Niš, Serbia; 8 Clinic and Policlinic for Dermatology and Venereology, University Medical Center Rostock, 18057 Rostock, Germany; ole.tiberius.jung@gmail.com

**Keywords:** 3D-printing, bioprinting, biphasic bone substitute, in vivo, macrophages, inflammation, bone regeneration

## Abstract

(1) Background: The aim of this study was examining the ex vivo and in vivo properties of a composite made from polycaprolactone (PCL) and biphasic calcium phosphate (BCP) (synprint, ScientiFY GmbH) fabricated via fused deposition modelling (FDM); (2) Methods: Scaffolds were tested ex vivo for their mechanical properties using porous and solid designs. Subcutaneous implantation model analyzed the biocompatibility of PCL + BCP and PCL scaffolds. Calvaria implantation model analyzed the osteoconductive properties of PCL and PCL + BCP scaffolds compared to BCP as control group. Established histological, histopathological and histomorphometrical methods were performed to evaluate new bone formation.; (3) Results Mechanical testing demonstrated no significant differences between PCL and PCL + BCP for both designs. Similar biocompatibility was observed subcutaneously for PCL and PCL + BCP scaffolds. In the calvaria model, new bone formation was observed for all groups with largest new bone formation in the BCP group, followed by the PCL + BCP group, and the PCL group. This finding was influenced by the initial volume of biomaterial implanted and remaining volume after 90 days. All materials showed osteoconductive properties and PCL + BCP tailored the tissue responses towards higher cellular biodegradability. Moreover, this material combination led to a reduced swelling in PCL + BCP; (4) Conclusions: Altogether, the results show that the newly developed composite is biocompatible and leads to successful osteoconductive bone regeneration. The new biomaterial combines the structural stability provided by PCL with bioactive characteristics of BCP-based BSM. 3D-printed BSM provides an integration behavior in accordance with the concept of guided bone regeneration (GBR) by directing new bone growth for proper function and restoration.

## 1. Introduction

The aim of bone regeneration is repairing a bony defect caused by a trauma, tumor, or disease. Bone augmentation can be achieved using biomaterials that function as an anchoring structure for new bone formation. These bone augmentation materials are divided into autografts, allografts, xenografts, and synthetic materials. Autografts are considered the golden standard for the process of Guided Bone Regeneration (GBR) due to the low risk of infection and risk of rejection [1] However, the use of autografts is strongly limited by its quantity and a high patient morbidity due to the requirement of an additional surgery site to obtain the bone material [1,2]. Due to the limited availability of autografts, allografts provide an alternative option of implanting human bone that is available in larger quantities. Nevertheless, the regenerative properties of allografts are lower, and the mechanical properties could alter [1]. Moreover, additional disadvantages of allografts are the potential for disease transmission and its costly storage [1,3]. Xenografts have similar properties to human bone, but their application may also include a high risk of an immunological reaction [1,4]. In contrast to natural bone grafts, synthetic materials can be provided in higher quantities with low risks of immunological reaction and rejection by the body [1]. Ceramic synthetic materials such as calcium phosphate (CaP) demonstrate excellent bioactivity and strong mechanical properties that make them ideal for use in bone tissue regeneration [5]. Different CaPs are available that vary in resorption rate and bioactivity [6]. Biphasic compounds of CaP composed of hydroxyapatite (HA) and beta-tricalcium phosphate (β-TCP) are already used in the daily clinical practice due to their adapted degradation behavior that has shown to be favorable for the process of bone regeneration [6,7]. However, difficulties have been described for producing pure CaP-based scaffolds for bone tissue regeneration due to their brittleness [8,9]. Thus, the possibilities of creating patient-individualized scaffolds from pure CaP are still limited in comparison to that of natural bone grafts, which can contain both mineral and collagen structures in a more durable structure [10].

Additive manufacture has the potential to produce composite scaffolds with a precise and well defined architecture [11]. Scaffolds can be created quickly without expensive and time-consuming post-processing [12]. Another advantage of 3D printing compared to traditional scaffold fabrication like electrospinning or freeze-drying, is the possibility of precise control over the inner architecture of the scaffold and the repeatability between scaffolds [13]. Moreover, it is possible to create patient-individual implants with the help of computer-aided design and manufacturing (CAD/CAM) [11]. Scaffolds can be produced out of various materials such as polymer, ceramics, and metals [1,14]. With respect to the wide range of materials, it is also possible to create composites combining ceramic particles in a polymeric matrix through additive manufacturing processes. Methods such as Fused Deposition Modeling (FDM) have become readily accessible for tissue engineering purposes due to the low setup costs and ease of application. FDM is a rapid printing method that extrudes thermoplastics or composite materials with a thermoplastic matrix, into predefined designs [15,16]. In the context of bone tissue regeneration, this printing method has already been used to synthesize biphasic 3D scaffolds composed of biologically resorbable polyesters and CaP-based bone substitutes [17,18,19]. It has been revealed that this combination promotes the desired cellular responses and bone regeneration [20,21,22,23].

It has yet to be established what effect these 3D printed composite materials have on the molecular or biological regenerative response, especially the material-associated inflammatory tissue response. Within the last decade it has been clarified that the inflammatory response to a biomaterial and specifically to bone substitute materials (BSM) is mainly dependent on the physicochemical material properties [24]. In this context, it has been revealed that macrophages, as well as their fused end stages, the multinucleated giant cells (MNGCs), are key elements in the tissue reaction that can direct the material-related regenerative fate due to their overall inflammatory alignment, i.e., the pro-inflammatory M1-phenotype or the anti-inflammatory M2-phenotype [25,26]. It is believed that materials that induce a more pronounced M2-reaction will most likely contribute to the process of tissue regeneration [27,28].

Thus, the aim of the present study was to examine the mechanical properties, biocompatibility, and the regenerative potential of newly developed 3D-printed scaffolds. These scaffolds were fabricated via FDM, combining polycaprolactone (PCL) used as a polymer matrix to support microparticles of a newly developed biphasic calcium phosphate (BCP) with a ratio of 80% PCL and 20% BCP. Established in vitro and in vivo analyses have been performed based on the international standard DIN ISO 10993 norm package. The in vivo study included two different implantation models (subcutaneous and calvaria implantation) in combination with specialized histological preparation methods, (immuno-) histochemical staining methods and specially designed histomorphometrical techniques [29].

## 2. Results

### 2.1. Ex Vivo Analysis

**Figure 1:**
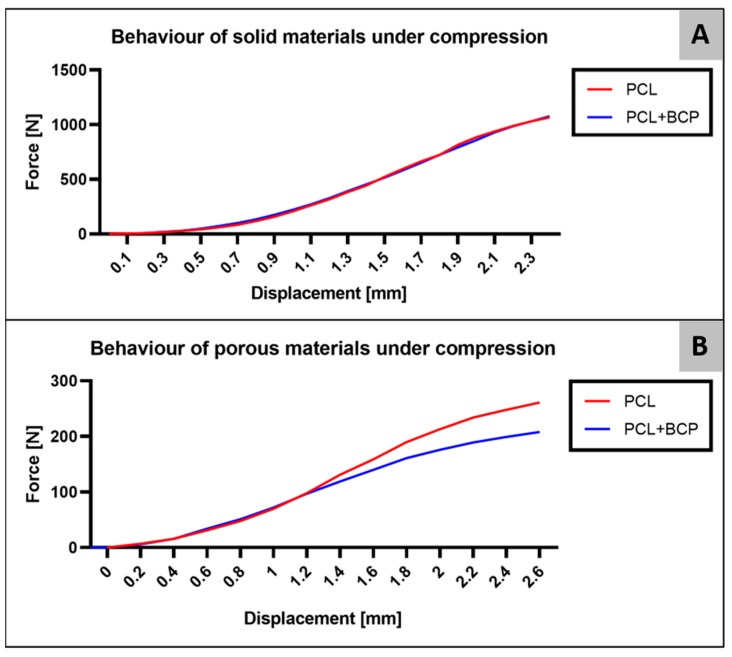
Force-displacement curves of (**A**) solid scaffolds and (**B**) porous scaffolds.

The compressive yield strength was considered as the yield point of the sample. The test continued until a yield point was observed where plastic deformation occurred, as is common in tests of ductile materials. Prior to this, linear deformation is observed, whereby the structure is continuously deformed until suspension of the applied load. The material’s behavior during this period is used for comparative purposes to determine the Young’s modulus and its proportionality to the yield. Some buckling was found in measuring cycle 1 for both porous scaffold designs, possibly due to some manufacturing flaws. Thus, graphs were made using the data from the force measuring cycles 2 and 3 for both materials. The data created from both measurement cycles can be observed in the force-displacement curves (Figure 1 and Table 1).

**Figure 2:**
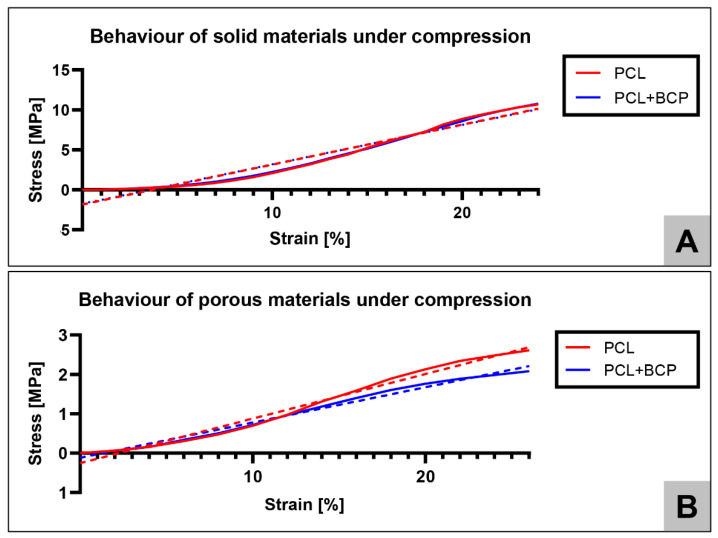
Stress-strain curves of (**A**) solid structures and (**B**) porous structures with dashed lines representing the linear regression curves.

The measurements revealed that the stress-strain curve of the scaffolds was non-linear (Figure 2). To support the cross-comparison of such experimental data with the linear FEA simulations, a single elastic yield strength and an elasticity Young’s modulus from the compression experiments (Table 2) was extracted. The yield strength modulus was defined as the ultimate stress perceived in the curve and the Young’s modulus as the slope of the stress-strain curve.

For the stress-strain tests curves, mathematical linear regression equations with a higher R-squared (R2 coefficient of determination) were found, being stress (Ss) and Strain (Sn). Graphs and mathematical equations were computed using the GraphPad Prism 9.0 software (GraphPad Software Inc., La Jolla, CA, USA).

**Figure 3:**
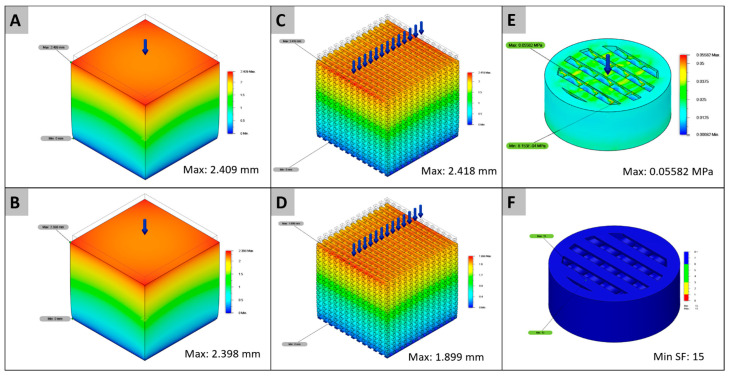
FEA Maximum displacements for PCL: (**A**) solid cubes and (**C**) porous cubes, PCL+BCP: (**B**) solid cubes and (**D**) porous cubes, (**E**) maximum stress for the implanted scaffold, and (**F**) minimum safety factor.

Then, using the loads for each displacement cases (Table 1) as entry data for the FEA prediction (Figure 3A–D) computational analysis results were validated against experimental data. (Table 3). The relative error was measured with the following equation (Equation (1)).

(1) $$\mathrm{Relative}\;\mathrm{Error}=\frac{\left|\mathrm{FEA}\;\mathrm{value}-\mathrm{real}\right|}{\mathrm{real}}\times 100\%$$

In the comparison between the mechanical tests and the FEA computational analysis (Table 3), similar values were calculated for the displacement of the solid PCL and PCL+BCP structures, as well as the porous PCL structure. However, both the porous structures had lower calculated displacement values than the mechanical essay. For the implanted scaffold designs, an equal von Mises maximum stress value of 0.05582 MPa and a minimum factor of 15 in both cases were calculated (Figure 3E,F).

### 2.2. Subcutaneous In Vivo Study

**Figure 4:**
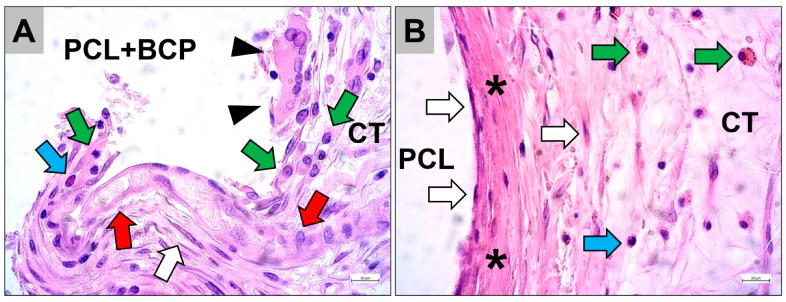
Representative images of the tissue reactions to the PCL scaffolds combined with the BCP granules (PCL+BCP) (**A**) and the pure PCL scaffolds (**B**) within the subcutaneous connective tissue (CT) at day 15 post implantationem. arrowheads = multinucleated giant cell, green arrows = macrophages, white arrows = fibroblasts, blue arrows = granulocytes, red arrows = blood vessel, asterisks = fibrotic capsule (HE-stainings, 400× magnifications, scalebars = 10 µm).

The histopathological observations at day 15 post implantationem revealed that both scaffold types were still present within the subcutaneous connective tissue and induced different tissue responses (Figure 4). At the material-tissue-interfaces of the PCL+BCP scaffolds, a cell- and vessel-rich granulation tissue was most often detected (Figure 4A). The granulation tissue included mainly macrophages alongside a smaller number of multinucleated giant cells (MNGCs) and fibroblasts in concert with a few granulocytes and lymphocytes (Figure 4A). In contrast, a slight fibrotic capsule with circularly arranged collagen fibers and mainly fibroblasts were found at the surfaces of the pure PCL scaffolds, while the neighbored connective tissue contained mainly macrophages, single granulocytes and lymphocytes (Figure 4B).

**Figure 5:**
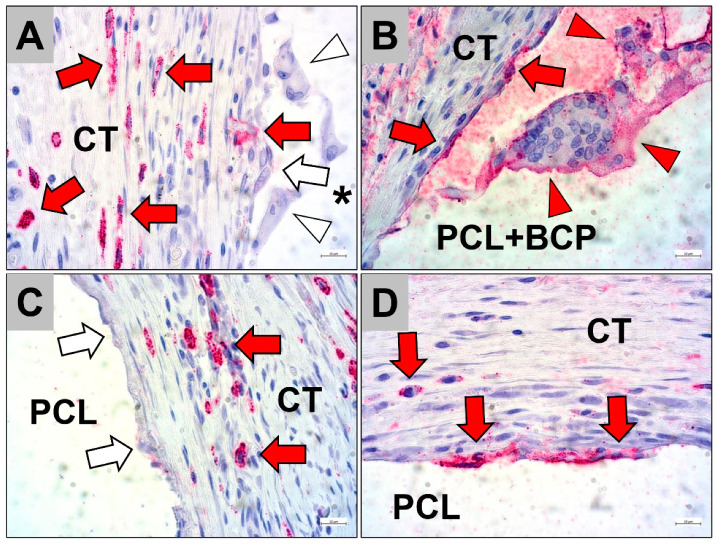
Representative images of the inflammatory tissue reactions to the scaffolds within the subcutaneous connective tissue (CT) at day 15 post implantationem. (**A**) CD163 detection and (**B**) CD11c detection within the implantation beds of the PCL scaffolds combined with the BCP granules (PCL+BCP and asterisk in (**A**)). (**C**) CD163 detection and (**D**) CD11c detection within the implantation beds of the pure PCL scaffolds. Red arrows = positive macrophages, white arrows = negative macrophages, red arrowheads = positive multinucleated giant cells, white arrowheads = negative multinucleated giant cells (immunostainings, 400× magnifications, scalebars = 10 µm).

The histopathological analysis of the occurrence of pro- and anti-inflammatory cells revealed comparably high numbers of anti-inflammatory CD163-positive cells within the implantation bed of both scaffold types (Figure 5). Whereby, CD163-positive cells were mainly found within the surrounding connective tissue of both scaffold types and also within the fibrous capsule adherent to the PCL scaffolds (Figure 5A,C). However, both the mononuclear and multinucleated cells that were directly attached to the material surfaces for both scaffold types often did not show any CD163 expression (Figure 5A,C).

Furthermore, the analysis showed that comparably lower numbers of CD11c-positive cells were observable within the implantation beds of both scaffold types (Figure 5). Moreover, slightly lower numbers of pro-inflammatory CD11c-positive cells were found within the implantation beds of the pure PCL scaffolds compared to that of the PCL+BCP-scaffolds (Figure 5). The histopathological analysis additionally revealed that a higher frequency of cells directly adherent to both material types expressed this molecule (Figure 5B,D). In case of the pure PCL scaffolds, only mononuclear cells were found to be CD11c-positive, while within the implantation beds of the PCL+BCP-scaffolds both mononuclear and multinucleated cells were CD11c-positive (Figure 5B,D).

**Figure 6:**
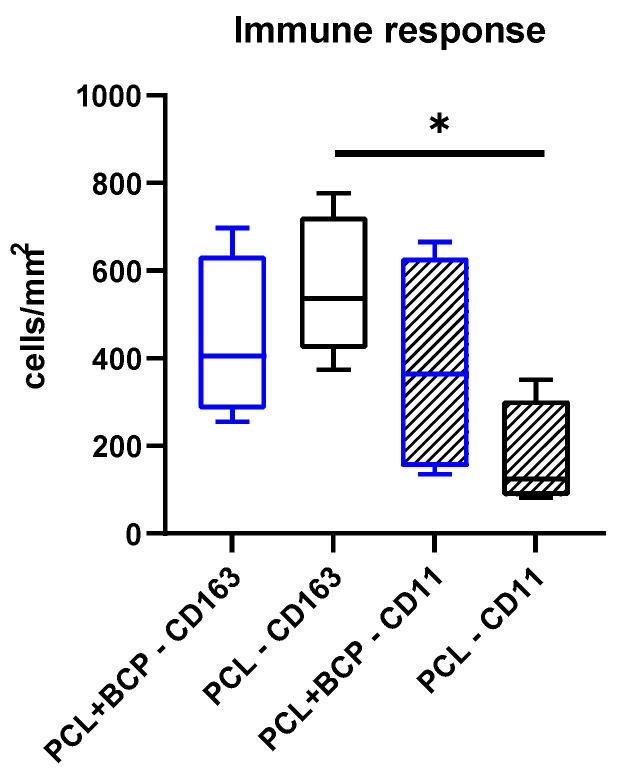
Histomorphometrical results of the measurements of the macrophage subtypes at day 15 post implantationem within the subcutaneous connective tissue (* = intraindividual differences, * *p* < 0.05).

The histomorphometrical analysis of the occurrence of M1- and M2-macrophages at day 15 post implantationem within the subcutaneous connective tissue revealed comparable numbers of both subtypes (Figure 6). Thus, in the PCL+BCP scaffolds group, 441.2 ± 187.6 CD163-positive cells per mm^2^ were measured compared to 565.3 ± 159.8 CD163-positive cells per mm^2^ in the PCL group. Further, in the PCL+BCP scaffold group 382.5 ± 252.5 cells per mm^2^ were measured and 180.7 ± 105.7 cells per mm^2^ were measured in the PCL scaffold group (Figure 6).

**Figure 7:**
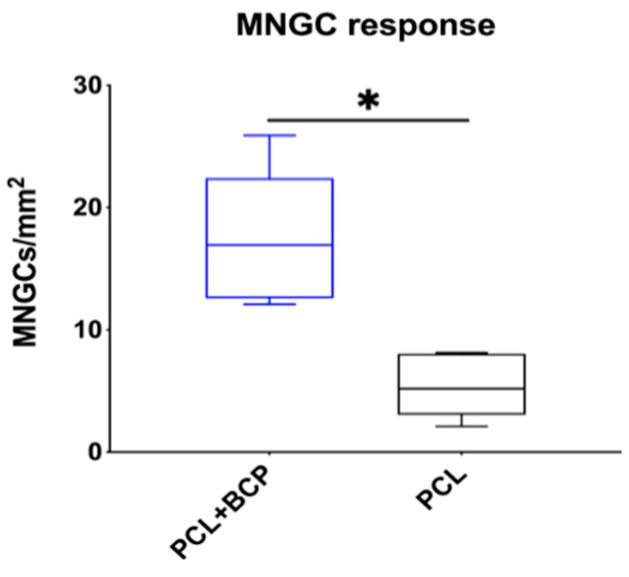
Histomorphometrical results of the measurements of the induction of multinucleated giant cells (MNGCs) at day 15 post implantationem within the subcutaneous connective tissue (* *p* < 0.05).

The analysis of the MNGC response further showed that significantly more multinucleated giant cells (17.39 ± 5.51 MNGCs/mm^2^) were found within the implantation beds of the PCL+BCP scaffolds (5.49 ± 2.59 MNGCs/mm^2^) compared to the numbers within the PCL-group (* *p* < 0.05) (Figure 7).

### 2.3. Calvarial In Vivo Study

**Figure 8:**
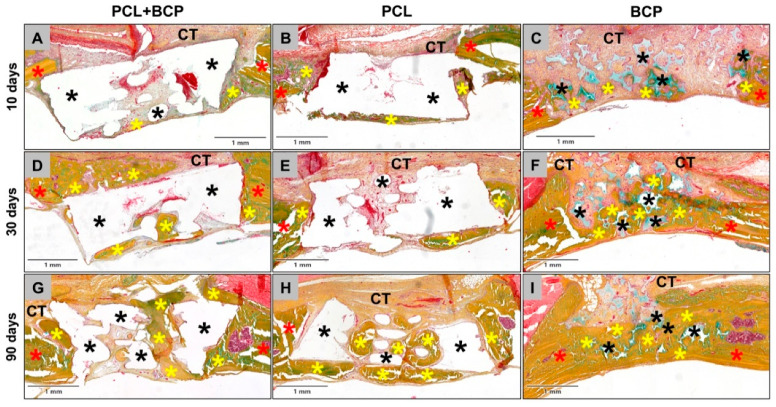
Exemplary total scans that show the (bone) tissue integration of the three biomaterials through the course of the study. (**A**–**C**) Implantations beds at day 10 post implantationem, (**D**–**F**) implantations beds at day 30 post implantationem and (**G**–**I**) implantations beds at day 90 post implantationem. Black asterisks = biomaterials, yellow asterisks = newly formed bone, red asterisks = local bone, CT = connective tissue (Movat Pentachrome stainings, 3.5× magnifications, scalebars = 1 mm).

The histological analysis of the bone healing process was performed with particular regard to the bony integration of the different biomaterials and their osteoconductive properties. At day 10 post implantationem, minimal bone growth onto the surfaces of all materials was detected, originating from the neighbored local calvaria bone (Figure 8A–C). For each study group, the materials were mainly surrounded by a cell- and vessel-rich connective tissue with a loose fiber density (Figure 8A–C). No visible differences to the amounts of newly formed bone tissue were observed between the different study groups at this early study time point (Figure 8A–C).

At day 30 post implantationem, the process of bony integration had comparatively increased in all three study groups, and nearly half of the implantation beds were filled by new bone tissue. Thus, in each study group, approximately half of the implanted biomaterial was integrated into a newly formed bone matrix (Figure 8D–F). The remaining implantation bed areas remained as a cell- and vessel-rich connective tissue (Figure 8D–F).

At day 90 post implantationem, the implantation beds of all three materials were mainly filled by newly formed bone tissue (Figure 8G–I). A nearly complete healing of the defect was almost only detectable in the group of the BCP granules, as the 3D-printed biomaterials were much more voluminous (Figure 8G–I). However, the observations showed that the fractions of the tissue areas seemed to contain comparable amounts of newly formed bone tissue. The remaining areas of the implantation beds remained as a cell- and vessel-rich connective tissue at this study time point (Figure 8G–I).

**Figure 9:**
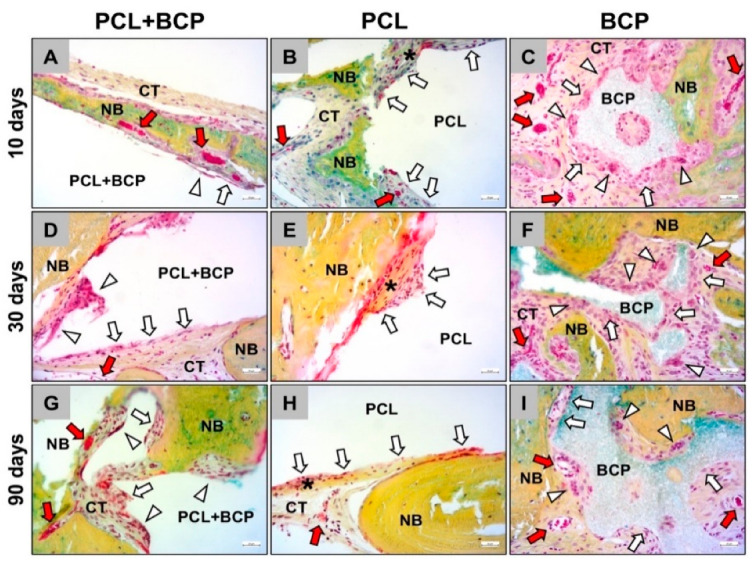
Exemplary histological images that show the tissue reactions to the three biomaterials through the course of the study. Tissue reactions at (**A**–**C**) day 10 post implantationem, (**D**–**F**) day 30 post implantationem and (**G**–**I**) day 90 post implantationem. White arrows = mononuclear cells, white arrowheads = multinucleated giant cells, red arrows = blood vessels, black asterisks = fibrosis-like tissue, NB = newly formed bone, CT = connective tissue (Movat Pentachrome stainings, 20× magnifications, scalebars = 20 µm).

The histopathological analysis revealed that all three materials were integrated within newly formed bone matrix over the study course, demonstrating osteoconductive properties (Figure 9). The surface areas of the materials that were covered by connective tissue, had mainly mononuclear cells and most often macrophages in case of the composite material PCL+BCP on their surface at all study time points (Figure 9A,D,G). In contrast, as was observed in the subcutaneous implantation model, a slight fibrotic capsule with circularly arranged collagen fibers and mainly fibroblasts were detectable at the surfaces of the pure PCL scaffolds, while mainly macrophages were found attached in many areas (Figure 9B,E,H). For the BCP, a slightly stronger inflammatory reaction was found locally restricted to the material surfaces, which involved mainly macrophages but also higher numbers of multinucleated giant cells (Figure 9C,F,I). All materials were also embedded into a cell- and vessel-rich connective tissue at all study time points (Figure 9). However, a higher vascularization was only observed within the implant beds of the PCL+BCP-scaffolds and the BCP granules (Figure 9).

**Figure 10:**
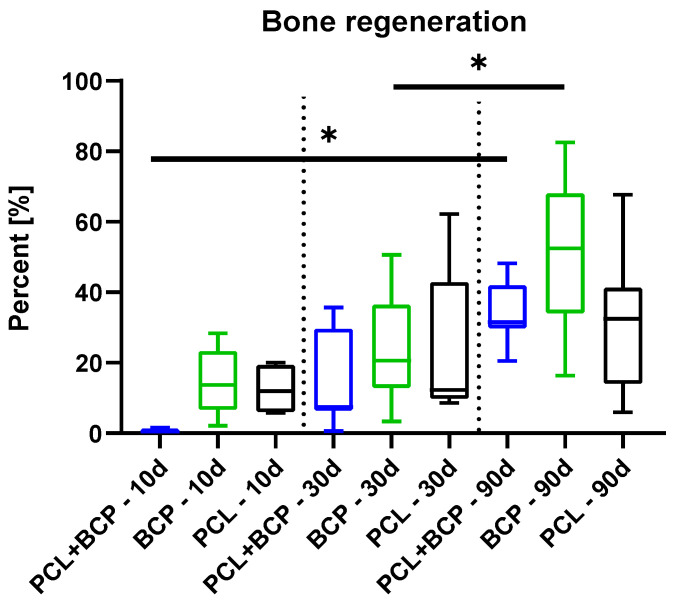
Histomorphometrical results of the measurements of bone regeneration (* = intraindividual differences, * *p* < 0.05).

The histomorphometrical analysis of bone formation revealed no significant differences between the three study groups at all of the study points (Figure 10 and Table 4). Only an intraindividual difference between the amounts at day 30 and 90 post implantationem in the BCP group (* *p* < 0.05) was detected (Figure 10 and Table 4).

**Figure 11:**
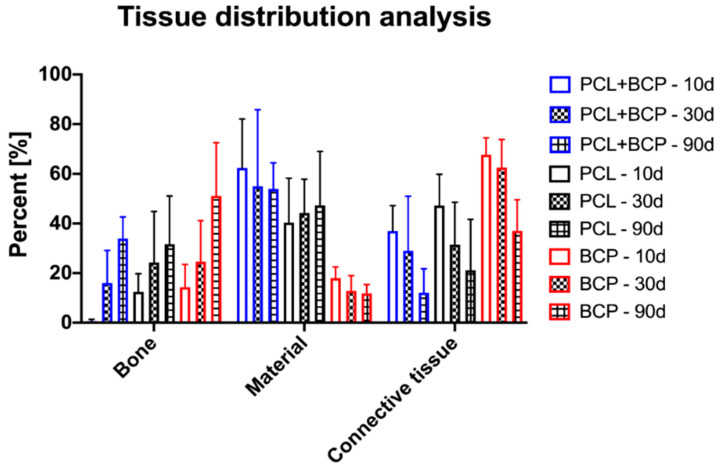
Histomorphometrical results of the tissue distribution measurements.

Histomorphometrical measurements at day 10 post implantationem showed that significantly more detectable biomaterial remained within the PCL+BCP-scaffold group compared to the BCP group (*** *p* < 0.001) (Table 4 and Figure 11). Furthermore, the analysis showed that significantly more biomaterial and connective tissue (CT) were found in the PCL+BCP-group compared to the amount of newly formed bone (** *p* < 0.01 and *** *p* < 0.001) (Table 4 and Figure 11). At this time point also more CT than newly formed bone was found in the PCL group (** *p* < 0.01) (Table 4 and Figure 11). In the BCP-group significantly more CT was found compared to amounts of newly formed bone and remaining biomaterial (*** *p* < 0.001) (Table 4 and Figure 11).

At day 30 post implantationem, significantly more biomaterial remained in the groups of the PCL+BCP- and the PCL-scaffolds compared to the BCP-group (** *p* < 0.01 and *** *p* < 0.001) (Table 4 and Figure 11). Additionally, significantly more CT was detected in the BCP-group compared to the amounts in the PCL+BCP- and the PCL-group (* *p* < 0.05) (Table 4 and Figure 11). In the PCL+BCP-group more biomaterial remained compared to the amount of newly formed bone tissue (** *p* < 0.01) (Table 4 and Figure 11). Finally, in the BCP-group significantly more CT compared to the amounts of newly formed bone and biomaterial was detected (** *p* < 0.01 and *** *p* < 0.001) (Table 4 and Figure 11).

At day 90 post implantationem, significantly more biomaterial remained in the groups of the PCL+BCP- and the PCL-scaffolds compared to the BCP-group (*** *p* < 0.001) (Table 4 and Figure 11). In the PCL+BCP-group, significantly more of the remaining biomaterial was detected compared to the amount of CT at this time point (*** *p* < 0.001) (Table 4). Finally, significantly more newly formed bone than remaining biomaterial was measured in the BCP-group (** *p* < 0.01) (Table 4 and Figure 11).

The analysis furthermore showed that the amounts of newly formed bone significantly increased between day 10 and day 90 post implantationem in the PCL+BCP- and the BCP-group (* *p* < 0.05 and ** *p* < 0.01) (Table 4 and Figure 11).

## 3. Discussion

The aim of the present study was to analyze the mechanical properties, biocompatibility and regenerative potential of newly developed 3D-printed scaffolds. These scaffolds were fabricated via FDM, combining polycaprolactone (PCL) as a polymer matrix material that incorporated biphasic calcium phosphate (BCP) microparticles that are used as a bone substitute material (BSM) with a proven biocompatibility. A loading weight ratio of 80% PCL and 20% BCP was used as it has already been reported that this ratio achieves the required biological and mechanical properties, as well as good workability [30,31,32].

In this context, PCL is a common biodegradable polymer used for a variety of medical applications and is especially suitable for 3D printing due to its low melting temperature of 55–60 °C [33]. Additionally, PCL has elastic properties at room temperature providing a degree of ductility not provided by purely ceramic scaffolds [34,35]. Due to it having a glass transformation temperature of −61 °C, its bulk properties should remain constant while in situ [33,36]. However, PCL has limited bioactivity and has a long degradation rate, sometimes remaining in situ years after implantation [37].

BCP is a combination of (β-TCP) and hydroxyapatite (HA) and has proven osteoconductive properties [7]. The composition of BCP make it ideal for bone regeneration, as the HA provides volume stability, whilst the β-TCP drives a bioactive response with its more rapid degradation [38]. It is similar to natural apatite, which is the main mineral constitute of human bone and is the main structural component that provides its rigidity [39]. With a high rate of osseointegration and volume stability, it can produce regenerative results comparable to xenografts [7,40,41]. The use of synthetic materials has the advantage over other grafts (e.g., natural BSM such as allo- or xenogeneic materials), as their material properties can be adjusted [42].

With respect to the chosen fabrication method, a composite made of PCL and BCP should combine the advantages of both material classes [43,44]. Previous studies have reported the combination of BCP and PCL creates a material with a high biocompatibility, mechanical stability, and a stable degradation. The BCP contributes a bioactive nature to the composite, forming a calcium-rich surface layer in situ [31,32,45].

Unlike the other manufacturing methods used for bone regeneration, such as solvent casting electrospinning or milling, FDM is a process that has complete control over scaffold shape and internal architecture. As it is an additive technique, there is also limited material wastage. Due to the CAD/CAM technique, scaffolds can be adapted to match the requirements of the implantation site, for example, by adjusting internal porosities to improve mechanical strength of scaffolds to be implanted in areas with high mechanical loading [15].

However, it is still unknown which molecular or biological influence these composite materials have on the bone regeneration process and the related material-associated inflammatory tissue response. Beside their osteoconductive properties, these materials should guide the bone repair process on the molecular level [46,47]. In this context, it is nowadays known that the inflammatory response to a resorbable BSM mainly includes macrophages and so-called multinucleated giant cells (MNGCs) as central elements of both the material-associated tissue regeneration process and the degradation process [47]. It was reported that biomaterials such as BSM should induced a more anti-inflammatory tissue response to stimulate (bone) tissue regeneration [24,48,49]. Simultaneously, it was shown that pro-inflammatory cells seem to be needed to process the material degradation that occurs, most often via phagocytosis by the afore mentioned mononuclear and multinucleated cell types [25,50]. Moreover, both cell types are also involved in different related cascades, such as the angiogenesis process by expression of signaling molecules, including the vascular endothelial growth factor (VEGF) [9,51].

Altogether, it was shown that all of these parameters are material-dependent as they are influenced by different physicochemical material properties, such as the chemical composition, the porosity, or BSM granule size [21]. Thus, the goal of any BSM development is to combine material factors to achieve a combined biological response that leads to an optimal bone tissue regeneration via appropriate biological responses in a specific indication or indication range. Hence, it is of particular importance to induce adapted cell responses that allow for a BSM application following the principle of “creeping substitution”, which means the near-complete resorption of the graft with simultaneous deposition of new, viable bone [52]. Finally, a resorbable BSM should optimally allow for a complete restitutio ad integrum in a specific application. Additionally, an optimal BSM has to provide different properties such as suitable mechanical properties for its application in load bearing defects [53,54].

It was initially shown in the present study that the FDM method enabled the creation of biphasic PCL+BCP-scaffolds with an adaptable architecture, which could be tailored to match the required structural properties, e.g., a pore size > 200 µm to promote bone tissue ingrowth, osteoblasts and vascularization [55,56,57]. Thus, this rapid manufacturing technique could be used to create scaffolds with unique shapes and configurations for bone tissue regeneration to structurally and mechanical fit into an individual defect site. This point is of special interest for bone tissue regenerative surgeries, as these scaffolds could eventually be produced in a patient specific manner to meet the individual regenerative requirements, replacing the conventional processes that are based on milling scaffolds out of natural bone blocks [58,59]. The use of CAD/CAM technology to manufacture patient specific BSM blocks using a milling process has already proved successful [60,61,62]. However, 3D-printed scaffolds are highly desirable due to the possibility to create not only a customized shape, but also control the internal scaffold architecture, favorable macro-micro-structure, hydrophilicity, mechanical strength and cellular responses [63].

The addition of a ceramic component to printed scaffolds can influence the material characteristics, such as changing the printing temperature or mechanical properties. Additionally, the configuration of a scaffold can also determine the scaffold mechanical properties, as already reported by other authors [64,65]. Based on the results obtained from mechanical tests in the current study, it can be concluded that in solid structures, both materials (PCL and PCL+BCP) behaved very similarly and present a viscoelastic behavior, since the stress-strain graphs showed a small initial progressive accumulation of stress followed by an approximately linear (elastic) response. Meanwhile, porous structures of these materials showed a more linear behaviour, demonstrating a mathematically linear progression, with a high value of R2 fits to model the stress-strain curve. Therefore, the porous structures more closely emulate the linear mechanical behaviour of bone [66]. In the case of the porous samples, much softer properties (approximately 5 times less) were calculated in comparison with the solid material samples (Table 2).

The different mechanical behaviour between the porous PCL and PCL+BCP structures might be associated with a decrease in layer adhesion caused by the addition of the BCP granules. The mean yield strength values obtained from the mechanical porous sample’s tests, i.e., 2.61 MPa for the PCL scaffolds and 2.08 MPa for the PCL+BCP scaffolds, are within the range for trabecular bone, as reported by Misch et al., where the compressive strength ranged from 0.22 to 10.44 MPa [67]. Altogether, the incorporation of BCP did not reduce the mechanical properties below that of trabecular bone. The porous scaffolds with a pore size (300 µm × 300 µm) makes them suitable for bone regeneration [65].

The FEA computational analysis results (Table 3) showed minimum error values in comparison between mechanical tests and FEA simulation for the solid samples, with an error of 0.37% for PCL and 0.08% for PCL+BCP. However, error values were higher when comparing the results in porous structures, with 7.00% for PCL and 26.96% for PCL+BCP. This increased error could be a consequence of a partial loss of layer adhesion in the manufacturing process, as previously mentioned for the cause in the differences in the stress-strain curves. For the prediction of the scaffold requirements inside the rat body, a value of 0.05582 MPa was used with a minimum safety factor of 15 (Figure 6) [68]. According to the simulation, the printed samples are suitable to be implanted and to hold the internal mechanical forces due to the systolic blood pressure inside the rat for the in vivo studies [68].

The results of the in vivo subcutaneous implantation model showed that both scaffold types were integrated within a cell and vessel-rich connective tissue without exaggerated tissue responses. Interestingly, the analysis revealed that the PCL+BCP-scaffolds induced a tissue response including macrophages and MNGCs at their material surfaces that seems to degrade the scaffolds, while the pure PCL scaffolds showed a more passivated tissue response inducing a slight fibrosis. This data could also be demonstrated within the calvaria bone tissue and lead to the conclusion that the combination of the PCL polymer with the BCP granules seems to combine the tissue reactions of each individual material, inducing a middle-grade inflammatory response that may end in an intermediate level of biodegradation. This conclusion is based on prior study results that showed that BCP materials combined the biological properties of the pure HA and the pure β-TCP group [7,38]. Thereby, the BCP materials initially induced a tissue reaction including high giant cell numbers comparable to the β-TCP-group, while the later tissue reaction was comparable to the HA-group. In conclusion, the combination of both compounds also resulted in a combined and balanced degradation pattern that forms the basis for successful bone tissue regeneration described for this material class [38]. The same tissue reaction pattern has been observed in the present study as the PCL+BCP scaffolds induced an inflammatory response, including MNGC numbers that are between those measured for the pure PCL scaffolds and the values found for the BCP granules reported in a previous study by Barbeck et al. using the same standardized implantation model [27,69,70,71,72]. Altogether, this data indicates that the combination of polymers with CaP-based BSM granules might be another possibility to guide the degradation behavior needed for bone tissue regeneration.

Additionally, the histomorphometrical measurements of the macrophage subtype numbers in the subcutaneous implantation model showed that comparable values of both pro- and anti-inflammatory cells were found within the implantation beds of both scaffold types at day 15 post implantationem, indicating that both materials seem to exhibit a similar level of biocompatibility. In this context, it has to be noticed that only the pure PCL scaffolds induced a more pronounced anti-inflammatory macrophage response, which may point to its better compatibility. However, the higher pro-inflammatory cell response in case of the PCL+BCP scaffolds correlate with the histological observations and also with the higher MNGC numbers. It was observed that the biomaterial-induced MNGCs that were significantly higher in the group of the PCL+BCP scaffolds only expressed the pro-inflammatory marker molecule CD11c. This observation substantiates a further presumption concluding that a pro-inflammatory tissue response including MNGCs mediates the degradation process of bone substitutes via phagocytosis as basis for a regeneration process following the principle of creeping substitution [72,73,74]. The authors finally assumed that even BSM inducing such a tissue reaction seem to be more favorable as they mediate a complete material resorption, which is needed for the regeneration process of bone tissue up to a restitution ad integrum [75,76].

The analysis of bone regeneration using the calvaria model additionally revealed that all materials induced comparable amounts of newly formed bone, guiding the bone growth process via osteoconductivity up to 90 days post implantationem. The highest amounts of bone at the latest study time point was found in the BCP group, and was preceded by a significant increase between day 30 and day 90. These results were not to be expected—especially since an implantation model with a critical size defects was chosen. But they are most likely explainable on basis of the demonstrated enormous healing potential of the chosen experimental animals [77,78]. The main focus, however, must be on the analysis of the tissue distribution, especially in the case of the analyzed BCP as a BSM with well-known regenerative capacities [7,79]. In this group the amount of newly formed bone was steadily increasing over time, while both the amounts of the remaining BSM and the connective tissue were decreasing. This healing process within the calvarial bone created a dense bone tissue area with low fractions of soft tissue. A continuous increase in bone growth (and also of the connective tissue) was also observed for both of the analyzed PCL and BCP+PCL scaffolds, however, the printed scaffolds had a significantly higher fraction of the BSM compared to the values found in the BCP group. In this instance, both scaffold types represented 3D-printed BSM “blocks” instead of the granules used in the BCP group. This led to an interesting tissue distribution tendency, whereby the amounts of the remaining BSM decreased slightly over the study period in the PCL+BCP-group comparably to the material fractions in the BCP group, while the fractions of the remaining BSM slightly increased over time. This phenomenon might be explainable based on the fact that is well known that PCL materials undergo a swelling process [80]. In this context, it has been reported that a swelling of PCL of about 10–20% occurs rapidly within the first 24 h [81]. Other studies showed that mixed materials, i.e., PCL mixed with hydrophilic biomaterials, decreased the swelling rate to 5–10% [82]. Thus, it is assumable that the analyzed scaffolds composed of PCL and BCP granules with a mix ration of 80 to 20% not only decreased the swelling behavior of the PCL, but led to the observed scaffold degradation. Additionally, these observations are in line with the observed changes in the tissue reactions that indicate a higher extent of cellular degradation in case of the PCL+BCP scaffolds.

However, the present study had limitations, which led to limited information, even regarding the tissue distribution and the degradation pattern of all analyzed materials. In this context, the application of another measurement techniques such as (synchrotron) µCT, nanoCT, µXRF and nanoXRF would provide deeper insights into the degradation behaviors of the biomaterials. Moreover, prolonged analysis of more complex periods are needed for the examination of the degradation behaviors of the BSM up to their complete resolution.

Altogether, the presented study results show that the newly developed PCL+BCP scaffolds are biocompatible and lead to a successful osteoconductive bone regeneration process. Interestingly, the combination of PCL with the biphasic BCP granules tailored the tissue responses towards a higher cellular biodegradability. Moreover, this material combination led to a reduced swelling, even compared to the pure PCL scaffolds. Altogether, the new biomaterial combines the volume stability of PCL with the bioactive characteristics of the BCP-based BSM. Thus, the 3D-printed BSM provides an integration behavior in accordance with the concept of guided bone regeneration (GBR) by mediating new bone growth at sites with insufficient volumes or dimensions of bone for proper function and restoration. It is possible that this integration pattern is also perfectly suitable for other clinical indications. Future research involving the bioprinting of the composite biomaterial presented in this article could include the deposition of living cells and other molecules such as extracellular matrix proteins, growth factors and exosomes during the printing procedure. This could increase the regeneration capacity of the construct [83]. Also, the biofabrication of multimaterial constructs with customized mesh structures will help to adapt the mechanical behavior of the scaffold to fit the mechanical performance of the anatomical part to be reconstructed.

## 4. Materials and Methods

### 4.1. Materials, Material Preparation and 3D-Printing

A homogenous composite material was fabricated with a composition of 80% PCL that had a MW 80,000 (Polysciences Europe, Hirschberg an der Bergstraße, Germany) and 20% of the synthetic biphasic calcium phosphate bone substitute material (BCP) (synprint, ScientiFY GmbH, Berlin, Germany) with a particle size of <40 µm.

**Figure 12:**
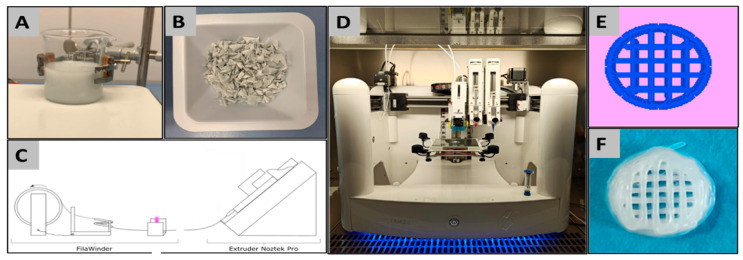
(**A**) PCL+BCP suspension, (**B**) PCL+BCP pellets, (**C**) schematic of Notztek Pro Extruder and FilaWinder assembly, (**D**) REG4Life Bioprinter, (**E**) scaffold design in REGEMAT designer V1.4.9, (**F**) 3D-printed scaffolds.

The composite material PCL+BCP made of PCL and BCP particles was fabricated by solvent casting using dichloromethane (Carl ROTH GmbH & Co.KG, Karlsruhe, Germany) to solve the PCL (Figure 12A) with constant stirring by a magnetic stirrer (IKA C-MAG HS 7, IKA^®^-Werke GmbH & Co. KG, Staufen, Germany). Under a fume hood, the solution was evaporated over a period of 24 h before being cut by hand into pellet form (Figure 12B). Printing filaments were produced by extrusion of the prepared pellets at 100 °C (Noztek Pro, Noztek, Shoreham, England) with a diameter of 1.75 mm that were collected and wound up on a spool (FilaWinder, Filastruder, Snellvile, Georgia, United States of America) (Figure 12C).

Scaffolds and test samples were designed using the REGEMAT Designer software 1.4.7 (Figure 12E) and manufactured for implantation and mechanical testing using a bioprinter (REG4Life, REGEMAT 3D, Granada, Spain) that included a heated platform and an extruder with a 0.4 mm nozzle (Figure 12D). The printing temperature was set at 120 °C for the PCL and 130 °C for PCL + BCP, the platform/ printing bed heated to 30 °C, and the printer speed set to 5 mms^−1^.

**Figure 13:**
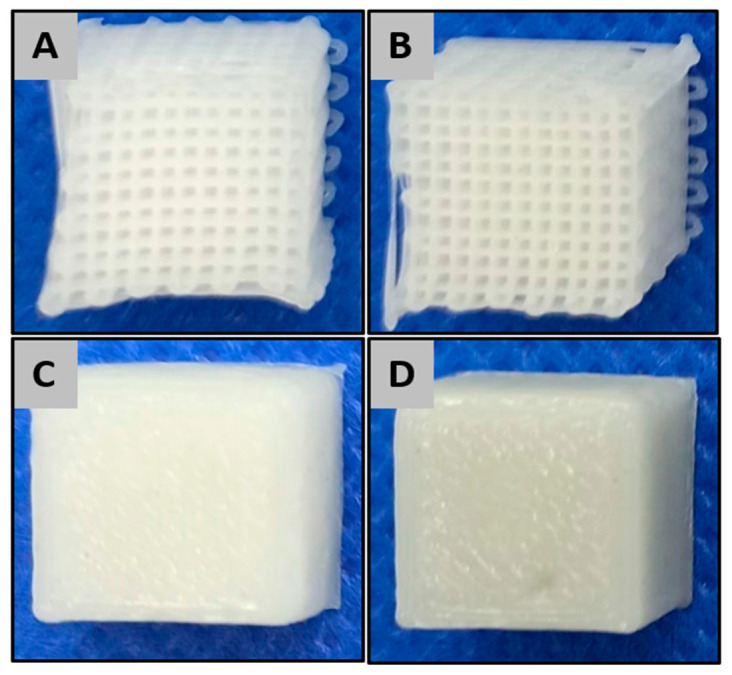
Scaffold design for mechanical testing. (**A**) porous structure of PCL scaffolds, (**B**) porous structure of PCL+BCP scaffold, (**C**) solid structure of PCL scaffolds, (**D**) solid structure of PCL + BCP scaffold.

For the mechanical tests of the materials, two sample configurations were designed and printed: the first, a solid cube and the second a porous mesh (Figure 13). Both sample designs had external dimensions of 0.4 mm × 0.4 mm and printed with a slicing height of 0.2 mm using alternate printing directions (0° and 90°) for each layer. For the implantation in vivo study in rats, 20 PCL and 20 PCL+BCP cylindric scaffolds were printed with a 3.9 mm ± 0.1 mm diameter and 1.5 mm ± 0.1 mm height. The scaffolds had a pore size of 300 × 300 µm (Figure 12F) (see Section 4.3).

### 4.2. Ex-Vivo Analyses

#### 4.2.1. Mechanical Properties

The sample’s mechanical properties were evaluated under compression using a MTS Model 835 Damper Test System uniaxial testing machine (MTS Systems Corporation, Eden Prairie, MN, USA) equipped with a 15 KN load cell. A constant strain rate of 0.01 mms^−1^ and 0.02 mms^−1^ with cubic mesh samples was used, adapted to the ISO 844 [84]. Three specimens (*n* = 3) were tested for each design. The force and the displacement were recorded throughout the test and thereafter converted to a stress versus strain curve. In the same way, the stress was defined as the mean measured force divided by the total area of the apparent cross section of the test sample, whilst the strain was evaluated as the ratio between the height variation and the initial height. Stress-strain curves were obtained from the data.

#### 4.2.2. Computational Analysis

**Figure 14:**
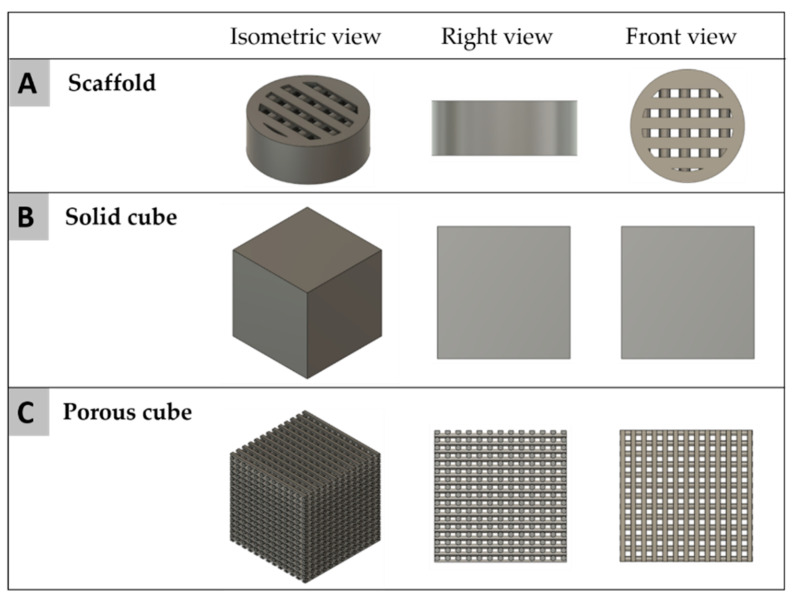
CAD design models of (**A**) in vivo scaffold, (**B**) solid cube, and (**C**) porous cube.

The mechanical response was studied through computational simulation. Test samples and scaffold geometries (Figure 14) were generated (Autodesk Fusion 360, Autodesk Inc., Mill Valley, CA, USA) and thereafter used for Finite Element Analysis (FEA) simulations. For the scaffold inside the body of the rat, a systolic pressure value of 120 mmHg (0.01599 MPa) was used [68]. Using the theoretical superficial area of the scaffold obtained by software as 9.19 mm^2^, a theoretical force (Equation (2)) of 0.1469 N was calculated and applied to perform the computational analysis.

The theoretical force was calculated using the following formula (Equation (2)) where P is the systolic pressure value of the rat and A is the theoretical superficial are of the implanted scaffold.

Force F = P × A F = 0.01599 MPa × 0.919 mm^2^ = 0.1469 N (2)

### 4.3. In Vivo Studies

Two in vivo studies were performed using previously established implantation models [27,69,70,71,72]. Initially, the subcutaneous implantation model was used to analyze the inflammatory tissue reactions to the newly developed scaffolds using both histopathological and histomorphometrical analysis methods. Additionally, the regenerative capacities of the scaffolds were examined after calvaria implantation.

#### 4.3.1. Implantation Procedures

Both in vivo studies were performed on 55 8–10 weeks old Wistar rats obtained from Military Medical Academy (Belgrade, Serbia) after the approval of the Local Ethical Committee (Faculty of Medicine, University of Niš, Niš, Serbia), based on decision number 323-07-00073/2017-05/7 of the Veterinary Directorate of the Ministry of Agriculture, Forestry and Water Management of the Republic of Serbia (date of approval: 22/02/2017). This study was conducted following the Animal Research Reporting In Vivo Experiment (ARRIVE) guidelines [85]. In total, a significant effect of 15% or more would be of interest. Considering previous rates for macrophage induction and new bone formation in the control group and biomaterial group(s) with significances of minimally 0.05 and power levels in accordance to the web site https://www.sealedenvelope.com/power/continuous-superiority/ (accessed on 25 March 2021), both study parts have considered to require a number of five animals per material/treatment and post-operative time point [39,48,70,71,72,73,74].

Animals were kept under standard conditions with regular mouse pellets, access to water ad libitum and an artificial light–dark cycle of 12 h each.

For the subcutaneous implantation model, 10 experimental animals with *n* = 5 animals per group, i.e., group 1: PCL+BCP and group 2: PCL, were used for one study time point (15 days). The subcutaneous implantations were conducted according to a previously established protocol by Barbeck et al. [27,71,86,87,88]. Briefly, an initial intraperitoneal anesthesia (10 mL ketamine [50 mg/mL] with 1.6 mL xylazine [2%]) was applied and the materials were implanted under sterile conditions in a prepared subcutaneous pocket within the animal’s subscapular region. Finally, the implantation wound was stitched with 5.0 Prolene (Ethicon US LLC, Cincinnati, OH, USA).

For the calvaria implantation model, 45 animals were randomly divided into three study groups, i.e., group 1: PCL+BCP, group 2: PCL and group 3: BCP, with *n* = 5 animals per group and study time point (10, 30 and 90 days). The established calvaria implantation was initiated by anesthesia using an intraperitoneal injection (10 mL ketamine (50 mg/mL) with 1.6 mL Xylazine (2%)) and following shaving and disinfecting of the implantation sites [89,90]. Afterwards, an incision in the skin was made and the muscle tissue covering the calvaria was prepared to create two calvaria defects (diameter: 5 mm) by means of a trephine bur, under local anesthesia with lidocaine (2%) and constant sterile saline irrigation. Subsequently, the biomaterials were inserted, and the wounds were sutured with suture material.

After euthanasia of the animals at the respective study time points by an overdose of ketamine and xylazine, tissue preparation was initiated by explantation of the biomaterials together with the surrounding tissue. Samples were fixated using 4% formalin for 24 h before their transfer into PBS until processed for histological workup.

#### 4.3.2. Histological Workup and Staining Methods

Dehydration of the samples was performed via a series of increasing alcohol concentrations and a final xylol treatment. For all samples from the implantation studies, plastic embedding in Technovit 9100 (Technovit 9100, Kulzer GmbH, Hanau, Germany) was conducted. A stepwise immersion at 4 °C with Technovit 9100 medium using different infiltration solutions (pre-infiltration, infiltration I + II with same composition) was conducted after dehydration, followed by polymerization according to the operation instructions. For successful polymerization, the explants were immediately stored at −20 °C until the liquid Technovit 9100 had completely hardened. Subsequently, the tissue blocks were trimmed into shape using a grinding machine (EcoMet 30, Buehler, Esslingen, Germany) and sections with a thickness of 4–6 µm were prepared using a rotation microtome (CUT4060E, microTec GmbH, Walldorf, Germany).

In both study parts, two histochemical stains, i.e., hematoxylin and eosin (HE), and Movat Pentachrome stains, were prepared for histological evaluation of the material-tissue-interactions. For the analysis of the inflammatory tissue response, the subcutaneous samples had two additional sections for every tissue explant used for immunohistochemical detection of pro- and anti-inflammatory macrophage subtypes. Two respective antibodies i.e., integrin alpha x (CD11c) (abx231412, Abbexa Ltd., Milton, UK) and hemoglobin scavenger receptor (CD163) (ab182422, abcam, Cambridge, UK) were used. The slides were initially treated with TRIS-EDTA pH 9 for 20 min in a steamer at 96 °C, followed by equilibration using a cold wash buffer. A blocking step with protein blocking solution for 10 min was conducted before incubation with the respective first antibody for 60 min at room temperature. Final detection of the antigen was caused by incubation with the biotinylated secondary antibody for 15 min, subsequently followed by application of the streptavidin–alkaline–phosphatase conjugate and the permanent AP-red chromogen. Finally, counterstaining was performed using Mayer’s hemalum solution (Merck KGaA, Darmstadt, Germany). Unless otherwise stated, all solutions and reagents were purchased from Zytomed Systems, (Berlin, Germany).

#### 4.3.3. Histopathological and Histomorphometrical Analysis Methods

The histopathological analysis was conducted to compare the tissue reactions to the different biomaterials based on a previously published protocol [29]. For this analysis, a light microscope Axio Scope.A1 (Carl Zeiss Microscopy GmbH, Jena, Germany) was used to evaluate the cells participating in the process of biomaterial integration and degradation, implantation bed vascularization, and possible adverse reactions, such as fibrotic encapsulation or necrosis. Histological figures were made using a microscope camera (Nikon DS-Fi1, Tokyo, Japan).

For the histomorphometrical analyses the areas of interest were initially digitized using a specialized scanning microscope, which consists of an Axio Scope. A1 microscope combined with an Axiocam 305 color digital camera and an automatic scanning table (Maerzhaeuser, Wetzlar, Germany) connected to a computer system running the ZEN Core software V3 (all: Zeiss, Oberkochen, Germany). The histomorphometrical analyses included measurements of the occurrence of pro- and anti-inflammatory macrophages and of the occurrence of MNGCs within the subcutaneous implant beds of the scaffold and the amounts of regenerated bone within the calvaria implant beds. For both analyses the respective total implantation areas of the implant beds were initially measured. To measure the numbers of M1- and M2-macrophage subforms and MNGCs, manual counting was conducted, and the corresponding densities were calculated (cells/mm^2^). For the calvaria study, the amounts of new bone were manually measured (in µm^2^) and related to the defect area, resulting in percentages of newly formed bone.

### 4.4. Statistical Analysis

The statistical analysis of the study data included an initial analysis of variance (ANOVA) and a following post-hoc LSD test by means of the GraphPad Prism 9.0 software (GraphPad Software Inc., La Jolla, CA, USA). Statistical differences were stated as significant if the *p*-values were below 0.05 (* *p* ≤ 0.05) and were considered highly significant if the *p*-values were less than 0.01 (** *p* ≤ 0.01) or even less than 0.001 (*** *p* ≤ 0.001). The quantitative data were finally graphed as mean ± standard deviation.

## Figures and Tables

**Table 1:** Force and displacement applied onto bulk and porous scaffold structures.

Mean Maximum Values	PCL (Solid)	PCL+BCP (Solid)	PCL (Porous)	PCL+BCP (Porous)
Force	1066 ± 88 N	1077 ± 35 N	261 ± 58 N	208 ± 25 N
Displacement	2.4 mm	2.4 mm	2.6 mm	2.6 mm

**Table 2:** Mechanical properties of PCL and PCL+BCP as solid and porous structure.

Mechanical Properties	PCL (Solid)	PCL+BCP (Solid)	PCL (Porous)	PCL+BCP (Porous)
Yield Strength	10.6600 MPa	10.7667 MPa	2.61 MPa	2.08 MPa
Young’s modulus	44.4167 MPa	44.8611 MPa	10.0385 MPa	8.00 MPa
Mathematical Equation	Ss = 0.4994 × Sn − 1.846	Ss = 0.4928 × Sn − 1.747	Ss = 0.1132 × Sn − 0.2551	Ss = 0.08961 × Sn − 0.1189
R^2^	0.9511	0.9565	0.9818	0.9878

**Table 3:** Comparison between FEA and mechanical essays of PCL and PCL+BCP as solid and porous structure.

Displacements	PCL (Solid)	PCL+BCP (Solid)	PCL (Porous)	PCL+BCP (Porous)
Mechanical Essay (Real)	2.4 mm	2.4 mm	2.6 mm	2.6 mm
Computational Analysis (FEA)	2.409 mm	2.398 mm	2.418 mm	1.899 mm
Relative error	0.37%	0.08%	7.00%	26.96%

**Table 4:** Results of the histomorphometrical tissue distribution measurements (in percent).

	10 Days			30 Days			90 Days		
	Bone	Material	CT	Bone	Material	CT	Bone	Material	CT
PCL+BCP	0.64 ± 0.76	62.40 ± 19.67	36.96 ± 19.18	13.84 ± 13.29	54.98 ± 30.81	41.91 ± 19.92	33.90 ± 8.73	58.96 ± 15.46	7.14 ± 16.39
PCL	12.45 ± 7.29	40.34 ± 14.87	47.22 ± 15.42	24.26 ± 20.57	44.28 ± 13.92	31.46 ± 15.22	31.61 ± 19.45	47.31 ± 19.03	21.08 ± 9.52
BCP	14.34 ± 9.13	17.95 ± 7.58	67.71 ± 4.81	24.64 ± 16.48	12.85 ± 5.61	65.59 ± 17.60	51.09 ± 21.45	11.84 ± 8.17	37.07 ± 18.19

## References

[1] Pawelec K.M. (2019). Introduction to the challenges of bone repair. Bone Repair Biomater..

[2] Stanovici J., Le Nail L.R., Brennan M.A., Vidal L., Trichet V., Rosset P., Layrolle P. (2016). Bone regeneration strategies with bone marrow stromal cells in orthopaedic surgery. Curr. Res. Transl. Med..

[3] Delloye C., Cornu O., Druez V., Barbier O. (2007). Bone allografts-What they can offer and what they cannot. J. Bone Jt. Surg..

[4] Skoglund A., Hising P., Young C. (1997). A clinical and histologic examination in humans of the osseous response to implanted natural bone mineral. Int. J. Oral Maxillofac. Implant..

[5] Arinzeh T.L., Tran T., Mcalary J., Daculsi G. (2005). A comparative study of biphasic calcium phosphate ceramics for human mesenchymal stem-cell-induced bone formation. Biomaterials.

[6] Jeong J., Kim J.H., Shim J.H., Hwang N.S., Heo C.Y. (2019). Bioactive calcium phosphate materials and applications in bone regeneration. Biomater. Res..

[7] Jelusic D., Zirk M.L., Fienitz T., Plancak D., Puhar I., Rothamel D. (2017). Monophasic ß-TCP vs. biphasic HA/ß-TCP in two-stage sinus floor augmentation procedures–a prospective randomized clinical trial. Clin. Oral Implant. Res..

[8] Karageorgiou V., Kaplan D. (2005). Porosity of 3D biomaterial scaffolds and osteogenesis. Biomaterials.

[9] Ghanaati S., Barbeck M., Orth C., Willershausen I., Thimm B.W., Hoffmann C., Rasic A., Sader R.A., Unger R.E., Peters F. (2010). Influence of β-tricalcium phosphate granule size and morphology on tissue reaction in vivo. Acta Biomater..

[10] Schnettler R., Franke J., Rimashevskiy D., Zagorodniy N., Batpenov N., Unger R.E., Wenisch S., Barbeck M. (2018). Allogenic Bone Grafting Materials-Update of the Current Scientific Status. Traumatol. Orthop. Russ..

[11] Tack P., Victor J., Gemmel P., Annemans L. (2016). 3D-printing techniques in a medical setting: A systematic literature review. Biomed. Eng. Online.

[12] Klocke F. (2015). Fertigungsverfahren 5-Geißen, Pulvermetallurgie, Additive Manufacturing.

[13] Hribar K.C., Soman P., Warner J., Chung P., Chen S. (2014). Light-assisted direct-write of 3D functional biomaterials. Lab Chip.

[14] Hänel T. (2008). Technologieentwicklung für die Herstellung patientenindividueller Knochenaufbauimplantate aus ß-Tricalciumphosphat durch 3D-Printing.

[15] Rider P., Kačarević Ž.P., Alkildani S., Retnasingh S., Schnettler R., Barbeck M. (2018). Additive Manufacturing for Guided Bone Regeneration: A Perspective for Alveolar Ridge Augmentation. Int. J. Mol. Sci..

[16] Ang T.H., Sultana F.S.A., Hutmacher D.W., Wong Y.S., Fuh J.Y.H., Mo X.M., Loh H.T., Burdet E., Teoh S.H. (2002). Fabrication of 3D chitosan-hydroxyapatite scaffolds using a robotic dispensing system. Mater. Sci. Eng. C.

[17] Sharma R., Singh R., Penna R., Fraternali F. (2018). Investigations for mechanical properties of Hap, PVC and PP based 3D porous structures obtained through biocompatible FDM filaments. Compos. Part B Eng..

[18] Jiao Z., Luo B., Xiang S., Ma H., Yu Y., Yang W. (2019). 3D printing of HA/PCL composite tissue engineering scaffolds. Adv. Ind. Eng. Polym. Res..

[19] Drummer D., Cifuentes-Cuéllar S., Rietzel D. (2012). Suitability of PLA/TCP for fused deposition modeling. Rapid Prototyp. J..

[20] Chen X., Gao C., Jiang J., Wu Y., Zhu P., Chen G. (2019). 3D printed porous PLA/nHA composite scaffolds with enhanced osteogenesis and osteoconductivity in vivo for bone regeneration. Biomed. Mater..

[21] Shim J.H., Moon T.S., Yun M.J., Jeon Y.C., Jeong C.M., Cho D.W., Huh J.B. (2012). Stimulation of healing within a rabbit calvarial defect by a PCL/ PLGA scaffold blended with TCP using solid freeform fabrication technology. J. Mater. Sci. Mater. Med..

[22] Konopnicki S., Sharaf B., Resnick C., Patenaude A., Pogal-Sussman T., Hwang K.G., Abukawa H., Troulis M.J. (2015). Tissue-engineered bone with 3-dimensionally printed β-tricalcium phosphate and polycaprolactone scaffolds and early implantation: An in vivo pilot study in a porcine mandible model. J. Oral Maxillofac. Surg..

[23] Bruyas A., Lou F., Stahl A.M., Gardner M., Maloney W., Goodman S., Yang Y.P. (2018). Systematic characterization of 3D-printed PCL/β-TCP scaffolds for biomedical devices and bone tissue engineering: Influence of composition and porosity. J. Mater. Res..

[24] Sridharan R., Cameron A.R., Kelly D.J., Kearney C.J., O’Brien F.J. (2015). Biomaterial based modulation of macrophage polarization: A review and suggested design principles. Mater. Today.

[25] Anderson J.M., Rodriguez A., Chang D.T. (2008). Foreign body reaction to biomaterials. Semin. Immunol..

[26] Anderson J.M., Jones J.A. (2007). Phenotypic Dichotomies in the Foreign Body Reaction. Biomaterials.

[27] Korzinskas T., Jung O., Smeets R., Stojanovic S., Najman S., Glenske K., Hahn M., Wenisch S., Schnettler R., Barbeck M. (2018). In vivo analysis of the biocompatibility and macrophage response of a non-resorbable PTFE membrane for guided bone regeneration. Int. J. Mol. Sci..

[28] Oishi Y., Manabe I. (2018). Macrophages in inflammation, repair and regeneration. Int. Immunol..

[29] Lindner C., Pröhl A., Abels M., Löffler T., Batinic M., Jung O., Barbeck M. (2020). Specialized Histological and Histomorphometrical Analytical Methods for Biocompatibility Testing of Biomaterials for Maxillofacial Surgery in (Pre-) Clinical Studies. In Vivo.

[30] Thuaksuban N., Pannak R., Boonyaphiphat P., Monmaturapoj N. (2018). In vivo biocompatibility and degradation of novel Polycaprolactone-Biphasic Calcium phosphate scaffolds used as a bone substitute. Biomed. Mater. Eng..

[31] Lei Y., Rai B., Ho K.H., Teoh S.H. (2007). In vitro degradation of novel bioactive polycaprolactone-20% tricalcium phosphate composite scaffolds for bone engineering. Mater. Sci. Eng. C.

[32] Wongsupa N., Nuntanaranont T., Kamolmattayakul S., Thuaksuban N. (2017). Assessment of bone regeneration of a tissue-engineered bone complex using human dental pulp stem cells/poly(ε-caprolactone)-biphasic calcium phosphate scaffold constructs in rabbit calvarial defects. J. Mater. Sci. Mater. Med..

[33] Nair L.S., Laurencin C.T. (2007). Biodegradable polymers as biomaterials. Prog. Polym. Sci..

[34] Xia Y., Zhou P.Y., Cheng X.S., Xie Y., Liang C., Li C., Xu S.G. (2013). Selective laser sintering fabrication of nano-hydroxyapatite/poly-ε-caprolactone scaffolds for bone tissue engineering applications. Int. J. Nanomed..

[35] Sharaf B., Faris C.B., Abukawa H., Susarla S.M., Vacanti J.P., Kaban L.B., Troulis M.J. (2012). Three-dimensionally printed polycaprolactone and β-tricalcium phosphate scaffolds for bone tissue engineering: An in vitro study. J. Oral Maxillofac. Surg..

[36] Guarino V., Gentile G., Sorrentino L., Ambrosio L., Mark H.F. (2017). Polycaprolactone: Synthesis, Properties, and Applications. Encyclopedia of Polymer Science and Technology.

[37] Dwivedi R., Kumar S., Pandey R., Mahajan A., Nandana D., Katti D.S., Mehrotra D. (2020). Polycaprolactone as biomaterial for bone scaffolds: Review of literature. J. Oral Biol. Craniofacial. Res..

[38] Ghanaati S., Barbeck M., Detsch R., Deisinger U., Hilbig U., Rausch V., Sader R., Unger R.E., Ziegler G., Kirkpatrick C.J. (2012). The chemical composition of synthetic bone substitutes influences tissue reactions in vivo: Histological and histomorphometrical analysis of the cellular inflammatory response to hydroxyapatite, beta-tricalcium phosphate and biphasic calcium phosphate cer. Biomed. Mater..

[39] Bal Z., Kaito T., Korkusuz F., Yoshikawa H. (2020). Bone regeneration with hydroxyapatite-based biomaterials. Emergent Mater..

[40] Ajanović M., Kamber-Ćesir A., Hamzić A., Tosum S. (2015). Measurements of Implant Stability Following Sinus Lift: A Pilot Clinical Study. Acta Stomatol. Croat..

[41] Di Raimondo R., Sanz-Esporrín J., Plá R., Sanz-Martín I., Luengo F., Vignoletti F., Nuñez J., Sanz M. (2020). Alveolar crest contour changes after guided bone regeneration using different biomaterials: An experimental in vivo investigation. Clin. Oral Investig..

[42] Langer R., Tirrell D.A. (2004). Designing materials for biology and medicine. Nature.

[43] Macedo F.A., Nunes E.H.M., Vasconcelos W.L., Santos R.A., Sinisterra R.D., Cortes M.E. (2012). A biodegradable porous composite scaffold of PCL/BCP containing Ang-(1-7) for bone tissue engineering. Ceramica.

[44] Causa F., Netti P.A., Ambrosio L., Ciapetti G., Baldini N., Pagani S., Martini D., Giunti A. (2006). Poly-ε-caprolactone/hydroxyapatite composites for bone regeneration: In vitro characterization and human osteoblast response. J. Biomed. Mater. Res. Part A.

[45] Thuaksuban N., Luntheng T., Monmaturapoj N. (2016). Physical characteristics and biocompatibility of the polycaprolactone-biphasic calcium phosphate scaffolds fabricated using the modified melt stretching and multilayer deposition. J. Biomater. Appl..

[46] Barrère F., van Blitterswijk C.A., de Groot K. (2006). Bone regeneration: Molecular and cellular interactions with calcium phosphate ceramics. Int. J. Nanomed..

[47] Barbeck M., Booms P., Unger R., Hoffmann V., Sader R., Kirkpatrick C.J., Ghanaati S. (2017). Multinucleated giant cells in the implant bed of bone substitutes are foreign body giant cells—New insights into the material-mediated healing process. J. Biomed. Mater. Res. Part A.

[48] Anderson J.M., Miller K.M. (1984). Biomaterial biocompatibility and the macrophage. Biomater. Silver Jubil. Compend..

[49] Brown B.N., Ratner B.D., Goodman S.B., Amar S., Badylak S.F. (2012). Macrophage polarization: An opportunity for improved outcomes in biomaterials and regenerative medicine. Biomaterials.

[50] Luttikhuizen D.T., Harmsen M.C., van Luyn M.J.A. (2006). Cellular and Molecular Dynamics in the Foreign Body Reaction. Tissue Eng..

[51] Tanneberger A.M., Al-Maawi S., Herrera-Vizcaíno C., Orlowska A., Kubesch A., Sader R., Kirkpatrick C.J., Ghanaati S. (2020). Multinucleated giant cells within the in vivo implantation bed of a collagen-based biomaterial determine its degradation pattern. Clin. Oral Investig..

[52] Athanasiou V.T., Papachristou D.J., Panagopoulos A., Saridis A., Scopa C.D., Megas P. (2010). Histological comparison of autograft, allograft-DBM, xenograft, and synthetic grafts in a trabecular bone defect: An experimental study in rabbits. Med. Sci. Monit..

[53] Amini A.R., Laurencin C.T., Nukavarapu S.P. (2012). Bone tissue engineering: Recent advances and challenges. Crit. Rev. Biomed. Eng..

[54] Damien C.J., Parsons J.R. (1991). Bone graft and bone graft substitutes: A review of current technology and applications. J. Appl. Biomater..

[55] Bai F., Wang Z., Lu J., Liu J., Chen G., Lv R., Wang J., Lin K., Zhang J., Huang X. (2010). The correlation between the internal structure and vascularization of controllable porous bioceramic materials in vivo: A quantitative study. Tissue Eng. Part A.

[56] Chen C.W., Betz M.W., Fisher J.P., Paek A., Chen Y. (2010). Macroporous hydrogel scaffolds and their characterization by optical coherence tomography. Tissue Eng. Part C Methods.

[57] Akay G., Birch M.A., Bokhari M.A. (2004). Microcellular polyHIPE polymer supports osteoblast growth and bone formation in vitro. Biomaterials.

[58] Khalyfa A., Vogt S., Weisser J., Grimm G., Rechtenbach A., Meyer W., Schnabelrauch M. (2007). Development of a new calcium phosphate powder-binder system for the 3D printing of patient specific implants. J. Mater. Sci. Mater. Med..

[59] Bose S., Vahabzadeh S., Bandyopadhyay A. (2013). Bone tissue engineering using 3D printing. Mater. Today.

[60] Blume O., Back M., Born T., Smeets R., Jung O., Barbeck M. (2018). Treatment of a bilaterally severely resorbed posterior mandible due to early tooth loss by Guided Bone Regeneration using customized allogeneic bone blocks: A case report with 24 months follow-up data. J. Esthet. Restor. Dent..

[61] Blume O., Donkiewicz P., Back M., Born T. (2019). Bilateral maxillary augmentation using CAD/CAM manufactured allogenic bone blocks for restoration of congenitally missing teeth: A case report. J. Esthet. Restor. Dent..

[62] Jacotti M., Barausse C., Felice P. (2014). Posterior atrophic mandible rehabilitation with onlay allograft created with cad-cam procedure: A case report. Implant Dent..

[63] Wang Y.-F., Wang C.-Y., Wan P., Wang S.-G., Wang X.-M. (2016). Comparison of bone regeneration in alveolar bone of dogs on mineralized collagen grafts with two composition ratios of nano-hydroxyapatite and collagen. Regen. Biomater..

[64] Majdzadeh-Ardakani K., Navarchian A.H., Sadeghi F. (2010). Optimization of mechanical properties of thermoplastic starch/clay nanocomposites. Carbohydr. Polym..

[65] Abbasi N., Hamlet S., Love R.M., Nguyen N.T. (2020). Porous scaffolds for bone regeneration. J. Sci. Adv. Mater. Devices.

[66] Oftadeh R., Perez-Viloria M., Villa-Camacho J.C., Vaziri A., Nazarian A. (2015). Biomechanics and Mechanobiology of Trabecular Bone: A Review. J. Biomech. Eng..

[67] Misch C.E., Qu Z., Bidez M.W. (1999). Mechanical Properties of Trabecular in the Human Mandible: Implications Dental Implant Treatment Planning and Surgical Placement. J. Oral Maxillofac. Surg..

[68] Gangwar A., Kumar P., Rawat A., Tiwari S. (2014). Noninvasive measurement of systolic blood pressure in rats: A novel technique. Indian J. Pharmacol..

[69] Barbeck M., Serra T., Booms P., Stojanovic S., Najman S., Engel E., Sader R., Kirkpatrick C.J., Navarro M., Ghanaati S. (2017). Analysis of the in vitro degradation and the in vivo tissue response to bi-layered 3D-printed scaffolds combining PLA and biphasic PLA/bioglass components–Guidance of the inflammatory response as basis for osteochondral regeneration. Bioact. Mater..

[70] Barbeck M., Motta A., Migliaresi C., Sader R., Kirkpatrick C.J., Ghanaati S. (2016). Heterogeneity of biomaterial-induced multinucleated giant cells: Possible importance for the regeneration process?. J. Biomed. Mater. Res. Part A.

[71] Barbeck M., Najman S., Stojanović S., Mitić Ž., Živković J.M., Choukroun J., Kovačević P., Sader R., James Kirkpatrick C., Ghanaati S. (2015). Addition of blood to a phycogenic bone substitute leads to increased in vivo vascularization. Biomed. Mater..

[72] Barbeck M., Udeabor S., Lorenz J., Schlee M., Holthaus M.G., Raetscho N., Choukroun J., Sader R., Kirkpatrick C.J., Ghanaati S. (2015). High-Temperature sintering of xenogeneic bone substitutes leads to increased multinucleated giant cell formation: In vivo and preliminary clinical results. J. Oral Implantol..

[73] Barbeck M., Lorenz J., Holthaus M.G., Raetscho N., Kubesch A., Booms P., Sader R., Kirkpatrick C.J., Ghanaati S. (2015). Porcine dermis and pericardium-based, non cross-linked materials induce multinucleated giant cells after their in vivo implantation: A physiological reaction?. J. Oral Implantol..

[74] McNally A.K., Anderson J.M. (2005). Multinucleated giant cell formation exhibits features of phagocytosis with participation of the endoplasmic reticulum. Exp. Mol. Pathol..

[75] Lorenz J., Barbeck M., Kirkpatrick C., Sader R., Lerner H., Ghanaati S. (2018). Injectable Bone Substitute Material on the Basis of ß-TCP and Hyaluronan Achieves Complete Bone Regeneration While Undergoing Nearly Complete Degradation. Int. J. Oral Maxillofac. Implant..

[76] Horch H.H., Sader R., Pautke C., Neff A., Deppe H., Kolk A. (2006). Synthetic, pure-phase beta-tricalcium phosphate ceramic granules (Cerasorb^®^) for bone regeneration in the reconstructive surgery of the jaws. Int. J. Oral Maxillofac. Surg..

[77] Gomes P.S., Fernandes M.H. (2011). Rodent models in bone-related research: The relevance of calvarial defects in the assessment of bone regeneration strategies. Lab Anim..

[78] Spicer P.P., Kretlow J.D., Young S., Jansen J.A., Kasper F.K., Mikos A.G. (2012). Evaluation of bone regeneration using the rat critical size calvarial defect. Nat. Protoc..

[79] Issa D.R., Abdel-Ghaffar K.A., Al-Shahat M.A., Hassan A.A.A., Iacono V.J., Gamal A.Y. (2020). Guided tissue regeneration of intrabony defects with perforated barrier membranes, simvastatin, and EDTA root surface modification: A clinical and biochemical study. J. Periodontal Res..

[80] Guvendiren M., Serpooshan V. (2020). 3D Printing for Tissue Engineering and Regenerative Medicine.

[81] Fadaie M., Mirzaei E. (2018). Nanofibrillated chitosan/polycaprolactone bionanocomposite scaffold with improved tensile strength and cellular behavior. Nanomed. J..

[82] Dimitriou R., Mataliotakis G.I., Calori G.M., Giannoudis P.V. (2012). The role of barrier membranes for guided bone regeneration and restoration of large bone defects: Current experimental and clinical evidence. BMC Med..

[83] Baena J.M., Jiménez G., López-Ruiz E., Antich C., Griñán-Lisón C., Perán M., Gálvez-Martín P., Marchal J.A. (2019). Volume-by-volume bioprinting of chondrocytes-alginate bioinks in high temperature thermoplastic scaffolds for cartilage regeneration. Exp. Biol. Med..

[84] International Organization for Standardization (2007). ISO 844:2007 (E): Rigid Cellular Plastics—Determination of Compression Properties.

[85] Kilkenny C., Browne W., Cuthill I.C., Emerson M., Altman D.G. (2010). Animal research: Reporting in vivo experiments: The ARRIVE guidelines. Br. J. Pharmacol..

[86] Willershausen I., Barbeck M., Boehm N., Sader R., Willershausen B., Kirkpatrick C.J., Ghanaati S. (2014). Non-cross-linked collagen type I/III materials enhance cell proliferation: In vitro and in vivo evidence. J. Appl. Oral Sci..

[87] Barbeck M., Lorenz J., Kubesch A., Bohm N., Booms P., Choukroun J., Sader R., Kirkpatrick C.J., Ghanaati S. (2015). Porcine dermis-derived collagen membranes induce implantation bed vascularization via multinucleated giant cells: A physiological reaction?. J. Oral Implantol..

[88] Barbeck M., Udeabor S.E., Lorenz J., Kubesch A., Choukroun J., Sader R.A., Kirkpatrick C.J., Ghanaati S. (2014). Induction of multinucleated giant cells in response to small sized bovine bone substitute (Bio-OssTM) results in an enhanced early implantation bed vascularization. Ann. Maxillofac. Surg..

[89] Sieger D., Korzinskas T., Jung O., Stojanovic S., Wenisch S., Smeets R., Gosau M., Schnettler R., Najman S., Barbeck M. (2019). The addition of high doses of hyaluronic acid to a biphasic bone substitute decreases the proinflammatory tissue response. Int. J. Mol. Sci..

[90] Flaig I., Radenković M., Najman S., Pröhl A., Jung O., Barbeck M. (2020). In vivo analysis of the biocompatibility and immune response of jellyfish collagen scaffolds and its suitability for bone regeneration. Int. J. Mol. Sci..

